# Comparative Investigation of Cellular Effects of Polyethylene Glycol (PEG) Derivatives

**DOI:** 10.3390/polym14020279

**Published:** 2022-01-11

**Authors:** Ha Pham Le Khanh, Dániel Nemes, Ágnes Rusznyák, Zoltán Ujhelyi, Pálma Fehér, Ferenc Fenyvesi, Judit Váradi, Miklós Vecsernyés, Ildikó Bácskay

**Affiliations:** 1 Department of Pharmaceutical Technology, Faculty of Pharmacy, University of Debrecen, Nagyerdei Körút 98, 4032 Debrecen, Hungary; pham.le.khanh.ha@euipar.unideb.hu (H.P.L.K.); nemes.daniel@pharm.unideb.hu (D.N.); rusznyak.agnes@pharm.unideb.hu (Á.R.); ujhelyi.zoltan@pharm.unideb.hu (Z.U.); feher.palma@pharm.unideb.hu (P.F.); fenyvesi.ferenc@pharm.unideb.hu (F.F.); varadi.judit@pharm.unideb.hu (J.V.); vecsernyes.miklos@pharm.unideb.hu (M.V.); 2 Doctorate School of Pharmaceutical Sciences, University of Debrecen, Nagyerdei Körút 98, 4032 Debrecen, Hungary; 3 Institute of Healthcare Industry, University of Debrecen, Nagyerdei Körút 98, 4032 Debrecen, Hungary

**Keywords:** Caco-2, MTT assay, NR assay, autophagy, flow cytometry, osmolality, *G. mellonella*

## Abstract

Nowadays, polyethylene glycols referred to as PEGs are widely used in cosmetics, consumer care products, and the pharmaceutical industry. Their advantageous properties such as chemical stability, low immunogenicity, and high tolerability explain why PEGs are applied in many fields of pharmaceutical formulations including parenteral, topical, ophthalmic, oral, and rectal preparations and also in modern drug delivery systems. Given their extensive use, they are considered a well-known group of chemicals. However, the number of large-scale comparative studies involving multiple PEGs of wide molecular weight range is low, as in most cases biological effects are estimated upon molecular weight. The aim of this publication was to study the action of PEGs on Caco-2 cells and *G. mellonella* larvae and to calculate the correlation of these effects with molecular weight and osmolality. Eleven PEGs of different molecular weight were used in our experiments: PEG 200, PEG 300, PEG 400, PEG 600, PEG 1000, PEG 1500, PEG 4000, PEG 8000, PEG 10,000, 12,000, and PEG 20,000. The investigated cellular effects included cytotoxicity (MTT and Neutral Red assays, flow cytometry with propidium iodide and annexin V) and autophagy. The osmolality of different molecular weight PEGs with various concentrations was measured by a vapor pressure osmometer OSMOMAT 070 and *G. mellonella* larvae were injected with the solutions of PEGs. Sorbitol was used as controls of the same osmolality. Statistical correlation was calculated to describe the average molecular weight dependence of the different measured effects. Osmolality, the cytotoxicity assays, flow cytometry data, and larvae mortality had significant correlation with the structure of the PEGs, while autophagosome formation and the proportion of early apoptotic cells showed no statistical correlation. Overall, it must be noted that PEGs must be tested individually for biological effects as not all effects can be estimated by the average molecular weight.

## 1. Introduction

Polyethylene glycol (PEG) polymers are an important group of excipients due to their widespread use in different pharmaceutical formulations. PEG refers to oligomers of polyethylene oxide with different molecular weight (MW) and they are commercially available in a wide range of MW from 200 to 10,000,000 g/mol [1,2]. Physical states are varied from the clear non-volatile liquid at room temperature (low molecular weight PEG: 200–700), through semisolid (pasty material, medium MW PEG: 800–2000), to solid (white waxy solid/flakes form/powder high MW: 3000 and above) [2,3].

PEGs earn their fame due to their non-immunogenicity, hydrophilicity, and good solubility improving capacity [3]. The terminal hydroxyl and ether groups contribute to the high polarity characteristic of PEGs [2,3]. The low molecular weight PEGs have more hydroxyl groups compared to their structure; thus, as the molecular weight of the PEGs increases, their solubility in water and other solvents decreases [2]. Moreover, PEGs can also be dissolved in many other solvents such as tetrahydrofuran, chloroform, dimethyl sulfoxide, and methanol [4]. Under normal conditions, PEGs can be considered relatively stable; the chemical and physical changes are limited and few. However, the interaction of PEGs with given compounds might induce precipitation such as phenol, cresol, resorcinol, and tannin, hence PEGs can be used as the antidote for removing certain toxic substances [5].

On their own, PEGs are widely applied as laxatives or in preparation for surgery or colonoscopy. Due to their ability to be miscible with aqueous fluids, PEGs are used as solubilizers and permeation enhancers for many poorly soluble and low permeability compounds (BCS class II, IV) thus significantly improving the bioavailability of the given drug. It was shown that the solubility of Diazepam and Temazepam was increased by 3.5-fold and 2.5-fold, respectively, at 30 °C in water [3]. An example of permeability improvement is that the Digoxin (BCS class IV) was shown to be absorbed much better in gastrointestinal tract when formulated in a PEG 400 solution encapsulated in soft gels (Lanoxincaps^®^) compared to a tablet dosage form (Lanoxin^®^) [2]. Moreover, some PEGs and its derivatives are also applied as penetration enhancers in topical dermatological preparations or cosmetics as surfactants, humectants, and emulsifiers [6]. PEG is often chosen to coat the particles surfaces of the specialized carriers used in targeted drug delivery system [7]. Due to the neutral, hydrophilic, and flexible features, these polymers form a surface layer on the nanoparticles for formulating stealth nanocarriers in cancer therapy. Thus, opsonin adhesion is reduced and the systemic circulation is prolonged by evading uptake by the reticuloendothelial system [3,8]. Doxorubicin, Taxol, and Camptothecin are typical examples of this PEGylation technique [3].

Despite their numerous advantages, several publications are available about the possible harmful effects of PEGs. Elevated serum osmolality, hypercalcemia, and renal failure were observed in an animal experiment in which New Zealand white rabbits with open wounds were treated topically with a PEG-based antimicrobial cream [9]. In a study involving Cynomolgus monkeys (*Macaca fascicularis*), pathological lesions in the kidney were observed at an oral dose of 2.2 to 4.4 g/kg PEG 200, during a 13-week treatment. Intratubular deposition of oxalate crystals were detected in the renal cortex [10]. Besides animal studies, there is also some evidence for PEG toxicity in humans. Three burned patients died following a treatment with a PEG-based burn cream (the cream contained 63% PEG 300, 5% PEG 1000, 32% PEG 4000, and approximately 0.01% of ethylene glycol) [11]. The autopsy of the patients revealed tubular necrosis which affected the proximal tube. Metabolic acidosis increased the serum calcium level and renal osmotic pressure. In summary, the main adverse effects of PEGs included metabolic acidosis, the increase in serum calcium, renal failure, and cytotoxicity [2,9,10,11].

In another study, PEG 4000, PEG 6000, and PEG 10,000 showed no cytotoxic effect on Caco-2 cells, while PEG 400 and PEG 15,000 at 4 *w*/*v*% exhibited significant toxicity on cells [12]. Parnaud et al. investigated the cytotoxicity of PEG 8000 on human adenocarcinoma HT29 and COLO 205 cells, the human foetal mucosa FHC cells and Caco-2 cells [13]. Their results showed that PEG 8000 was not significantly toxic on Caco-2 and FHC cells, but it severely inhibited the proliferation of HT29 and COLO205 cells. They suggested that PEG 8000 might own a selectively cytostatic effect on proliferating cancer cells due to its high osmotic effect.

We aimed to study the correlation between cell viability, autophagosome formation, in vivo toxicity, and the molecular weight. As most studies involved only a limited number of derivatives, and we wished to better understand the cytotoxicity of these compounds, we investigated eleven substances of various molecular weights on a much wider scale: PEG 200, PEG 300, PEG 400, PEG 600, PEG 1000, PEG 1500, PEG 4000, PEG 8000, PEG 10,000, PEG 12,000, and PEG 20,000.

## 2. Materials and Methods

### 2.1. Materials and Sample Preparation

A total of 12 polyethylene glycols derivatives were chosen to be investigated based on their average molecular weight. Range of molecular weight is also indicated. PEG 200 (190–210 MW), PEG 300 (290–305 MW), and PEG 600 (550–650 MW) were obtained from TCI (Zwijndrecht, Belgium). PEG 1000 (950–1050 MW), PEG 8000 (7000–9000 MW), PEG 10,000 (9000–11,250 MW), PEG 12,000 (11,000–13,000 MW), and PEG 20,000 (16,000–24,000 MW) were purchased from Alfa Aesar (Karlsruhe, Germany). PEG 400 (380–420 MW), PEG 1500 (1400–1600 MW), PEG 4000 (3500–4500 MW), and sorbitol were obtained from Molar Chemicals (Halásztelek, Hungary). All the PEGs were stored under dry and cool conditions.

The 3-(4,5-dimethylthiazol-2-yl)-2,5-diphenyltetrazolium bromide (MTT), sodium chloride, Dulbecco’s Modified Eagle’s Medium (DMEM), phosphate-buffered saline (PBS), trypsin from porcine, EDTA, heat-inactivated foetal bovine serum (FBS), and propidium iodide solution were purchased from Sigma-Aldrich (Budapest, Hungary). Lonza (Basel, Switzerland) provided the non-essential amino acid solution and penicillin-streptomycin mix. GlutaMax™ supplement and Annexin V Alexa Fluor™ 647 conjugate were purchased from Thermo Fisher (Budapest, Hungary). Alfa Aesar (Karlsruhe, Germany) product was the Neutral Red (3-amino-7-dimethylamino-2-methylphenazine hydrochloride). Enzo Life Sciences (Farmingdale, NY, USA) provided the CYTO-ID Autophagy Detection Kit.

The PEGs with different molecular weights were all measured. PEGs were dissolved in PBS at 30 *w*/*v*% concentration and these solutions were used for all experiments. All of the PEG test solutions were freshly prepared immediately before any given experiment. Different sorbitol solutions were also dissolved with PBS and freshly prepared.

### 2.2. Cell Culture Maintenance

The Caco-2 cell line were purchased from the European Collection of Cell Cultures (Salisbury, United Kingdom—catalogue No. 8601020). Cells were cultured in Nunc™ EasyFlask™ cell culture flasks (Thermo Fisher, Darmstadt, Germany) in DMEM (containing: 0.584 g/L L-glutamine and 4.5 g/L D-glucose), supplemented with 10% (*v*/*v*) FBS, 3.7 g/L sodium hydrogencarbonate, 1% (*v*/*v*) non-essential amino acid solution, and 100 IU/mL penicillin K, with 100 μg/mL streptomycin sulfate at 37 °C in an atmosphere of 5% CO_2_. Passaging was regularly performed for cell maintenance and fresh glutamine was continuously supplemented by GlutaMax™. Our experiments were carried out on cultures between passage numbers 25–40 [14].

### 2.3. Cytotoxicity Assays

The cytotoxic effects of all PEG and sorbitol solutions were measured by MTT and Neutral Red (NR) assays. Caco-2 cells with media were seeded into 96-wells plates (VWR International Inc., Debrecen, Hungary) at a density of 1 × 10^4^ cells/well. After 7 days, the medium was removed, and the cells were treated with 100 µL of the test solutions for 30 min at 37 °C. Concentrations of the test solutions were 30 *w*/*v*% for all the PEG solutions and 7–10–12.5–35 *w*/*v*% for the sorbitol solutions. During the preliminary studies, lower concentrations of PEG solutions were also tested, but cytotoxicity did not exceed 20%. In case of MTT assay, the test solutions were removed and a 0.5 mg/mL MTT solution (PBS as a solvent) was added to each well. The plate was again incubated for 3 h at 37 °C. In case of Neutral Red assay, a 16.6 mg/mL NR solution (cell culture medium as a solvent) was added to each well after removing all test solutions. The plate was incubated for 2 h in this case. After incubation time, all dyes were completely removed and 0.1 mL of an isopropanol-1 M hydrochloride acid (25:1) solution was added to each well to dissolve the cells and solubilize the formazan crystals and the incorporated Neutral Red. The absorbance of the wells was measured at 565 nm for MTT assay and 540 nm for NR assay. We used empty wells of the plate as a background and all the measurements were carried out with a Thermo-Fisher Multiskan Go (Thermo-Fisher, Waltham, MA, USA) microplate reader. Cell viability was expressed as a percent of the cell viability of the untreated control cells, which were incubated with PBS for 30 min in case of both methods [14].

### 2.4. Osmolality Measurement

The OSMOMAT 070 vapor pressure osmometer (Gonotec GmbH, Berlin, Germany) is suitable for directly determining the total osmolality of aqueous solutions. The measurement temperature was 45 °C and the sampling time was 5 min. The solvent chosen was ultrapure (Type 1) water obtained from a Millipore Direct-Q 5 UV system (Millipore SAS, Molsheim, France). Before each experiment, the baseline was determined by ultrapure water. After the baseline was stable, the calibration was carried out with a 1 *w*/*v*% sodium chloride solution. After the cell constant was calculated and the system calibrated with it, the PEG and sorbitol samples with increasing concentration were measured sequentially. All liquids were dropped upon the sensors twice and the second drop was used for measurement. Concentrations of the test solutions were 30 *w*/*v*% for all the PEG solutions and 7–10–12.5–35 *w*/*v*% for the sorbitol solutions. The osmolality of the samples was expressed in mOsmol/kg, as an average of 4 individual measurements.

### 2.5. Flow Cytometry Analysis

A Guava^®^ easyCyte™ 5HT (Luminex, Austin, TX, USA) flow cytometer was used for our experiments; 3 × 6.5 million Caco-2 cells were collected from cell culture flasks with a trypsin-EDTA solution and redistributed into separate tubes and 1 million cells were treated with 1 mL of PEG test solutions dissolved in PBS. Concentration of the test solutions was 30 *w*/*v*% for all the PEG solutions. After 30 min, the cells were centrifuged, the test solutions were removed, and the cells were gently washed with cold PBS and centrifuged again. Supernatant was removed and with annexin-binding puffer a 1 million cells/mL cell suspension was created. Then, 100 µL of these suspensions were treated with 5 µL of Alexa Fluor™ 647 and 1 µL of 100 µg/mL propidium iodide solution. The cell suspensions were stained for 15 min on ice and then distributed on a 96-well microplate (3 wells/group) and analyzed. The propidium iodide was excited with a 488 nm laser and detected at the 525/30 nm channel (green parameter). The Alexa Fluor™ 647 was excited with the same laser line and detected at the 695/50 nm channel (red parameter). On the FSC-SSC scatterplot the non-cellular events were excluded. On the FSC-A-FSC-W scatterplot the duplets were excluded. The remaining events (8000–10,000) were analyzed on a propidium iodide-Alexa Fluor 647 scatterplot, the quadrant gates were determined on non-labelled samples. The double positive cells were regarded as necrotic/late apoptotic cells. The annexin V positive population was regarded as early apoptotic, the double negative population regarded as viable cells [14].

### 2.6. Autophagy Assay

The quantitative measurement of autophagosomes was carried out the CYTO-ID Autophagy Detection Kit (Enzo Life Sciences, Farmingdale, NY, USA) which is based on the staining autophagosomes. Caco-2 cells in the density of 10,000 cells/well were seeded into black 96-well plates (Greiner Bio-One, Mosonmagyaróvár, Hungary). When cells reached the appropriate confluence on the microplates (4 days), they were incubated for 30 min with 100 µL of the PEG and sorbitol test solutions at 37 °C. Concentrations of the test solutions were 30 *w*/*v*% for all the PEG solutions and 7–10–12.5–35 *w*/*v*% for the sorbitol solutions. After the treatment, wells were washed once with PBS. Cells were incubated with 1 mL of the given buffer which was supplemented with 1 µL CYTO-ID^®^ Green Detection Reagent and 1 µL Hoechst 33,342 Nuclear Stain for 30 min at 37 °C. After another washing with PBS, green fluorescence intensities of the samples were measured with FLUOstar Optima microplate reader (BMG Labtech, Offenburg, Germany) at 485 nm excitation and 520 nm emission wavelengths. Hoechst 33,342 Nuclear Stain was measured at 365 nm excitation and 445 emission wavelengths. According to the manufacturer’s specification, green fluorescence values were normalized to the blue fluorescence values [15].

### 2.7. G. mellonella Larvae Survivability Tests

Bugs World Inc. (Budapest, Hungary) provided the larvae of the sixth developmental stage of *G. mellonella*. Before the treatment, all larvae were kept at 10 °C and in a dark environment. Specimens with length between 2 and 3 cm and lacking any sign of melanisation were placed in sterile vented Petri dishes. Next, 20 µL of the test solutions were injected into the specimens around their last pro-leg with a 29 G needle. Concentration of the test solutions was 30 *w*/*v*% for all the PEG solutions. After treatment, the larvae were kept at 30 °C for 48 h shielded from light. Viability was regularly checked by gentle probing with a blunt-ended needle. If no movement was detected after the probing, the given larva was considered to be dead. Viability was observed at 19 h, 24 h, 43 h, and 48 h [14].

### 2.8. Statistical Analysis

Statistical data analysis and plotting were carried out with GraphPad Prism software (version 8; GraphPad Software, San Diego, CA, USA). All data were presented as means ± SEM. In case of the cell viability assays, each column represents the mean of ten independent, parallelly treated wells. PEGs and sorbitol treated cells’ absorbance values were compared with their given control group as they were measured on multiple microplates. Gaussian distribution was analysed with Shapiro–Wilk test and equal variances with Bartlett’s test. If the data set passed both tests, a one-way ANOVA was calculated, if Bartlett’s test failed, a Welsch’s ANOVA was calculated, and if Gaussian distribution was not proven, then a Kruskal–Wallis test was carried out. As a post test, Dunnett’s test was used to compare the treated cells’ results to the controls. In each case we used significance level *p* < 0.05. Significance is labelled as ns = *p* ≥ 0.05; * = *p* < 0.05; ** = *p* < 0.01; *** = *p* < 0.001; and **** = *p* < 0.0001. In vivo survival curves of *G. mellonella* larvae were plotted on Kaplan–Meier curves and the different groups’ survival was compared with Mantel–Cox log-rank test and Gehan–Breslow–Wilcoxon test. Osmolality, autophagy, and flow cytometry results were not analysed due to low number of parallel experiments (n = 4, n = 4, and n = 3, respectively). Due to this, we used Spearman correlation to calculate the relationship between molecular weight and other measured data (*p* < 0.05, two-tailed).

## 3. Results

### 3.1. Osmolality Results

**Figure 1:**
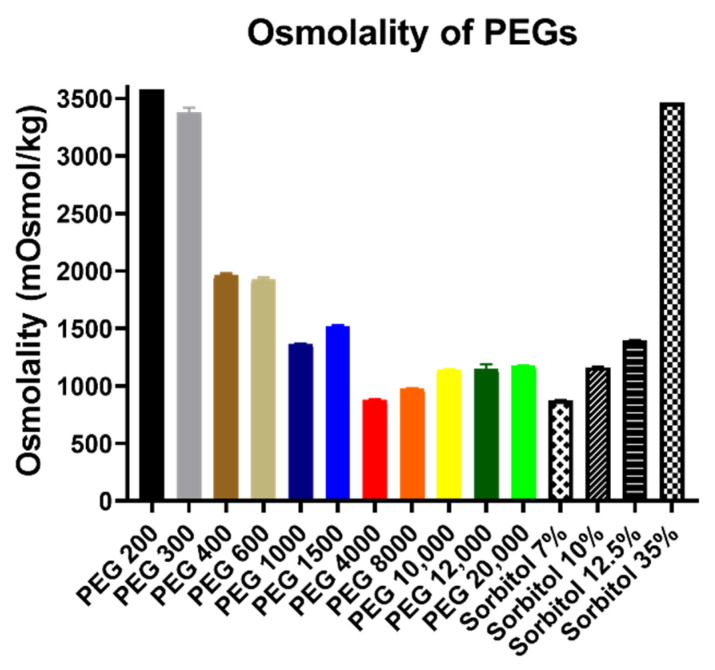
Osmolality of PEG and sorbitol solutions measured by vapor pressure osmometer OSMOMAT 070. Concentrations of all PEG solutions were 30 *w*/*v*% and volumes were 1 drop of the solutions each time. Columns represent the mean, ± SEM, n = 4. Osmolality of the samples in mOsmol/kg (mean ± SEM): PEG 200: 3574 ± 0; PEG 300: 3381 ± 38; PEG 400: 1963 ± 17; PEG 600: 1926 ± 17; PEG 1000: 1363 ± 4; PEG 1500: 1515 ± 11; PEG 4000: 877 ± 9; PEG 8000: 977 ± 3; PEG 10,000: 1140 ± 4; PEG 12,000: 1151 ± 38; PEG 20,000: 1174 ± 5; Sorbitol 7 *w*/*v*%: 876 ± 2; Sorbitol 10 *w*/*v*%: 1164 ± 1; Sorbitol 12.5 *w*/*v*%: 1395 ± 4; and Sorbitol 35 *w*/*v*%: 3464 ± 0.

At first, the osmolality of PEGs was defined at 30 *w*/*v*%. Based upon our preliminary research, the cytotoxicity of PEGs was negligible (cell viability was above 80% for all derivatives) at concentrations below 30 *w*/*v*%. In order to let all derivatives exhibit their full effect on cells and in vivo specimens, further increase in concentration was needed. Sorbitol solutions were also tested in order to distinguish which biological properties are affected by osmolality and which are not. Figure 1 shows that osmolality drastically decreased from PEG 200 until PEG 4000. From this derivative, the values minimally increased until PEG 10,000 and above when values were similar.

### 3.2. Cytotoxicity Assay Results

**Figure 2:**
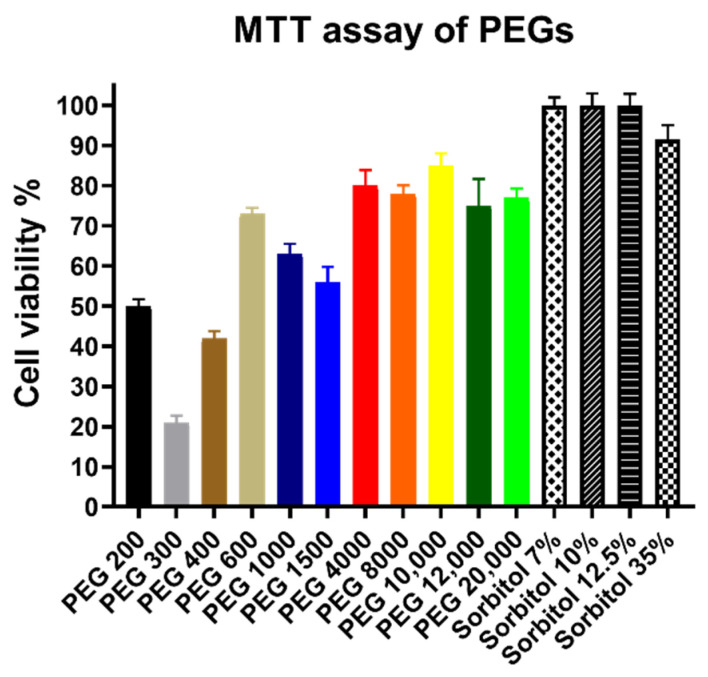
Cytotoxicity of PEG and sorbitol solutions measured by MTT assay on Caco-2 cells. Concentrations of all PEG solutions were 30 *w*/*v*% and volumes were 100 µL. Cell viability expressed as the percentage of the absorbance of the untreated control cells. Columns represent the mean, ± SEM, n = 10. Cell viability of the samples (mean ± SEM): PEG 200: 50% ± 2%; PEG 300: 21% ± 2%; PEG 400: 42% ± 2%; PEG 600: 73% ± 2%; PEG 1000: 63% ± 3%; PEG 1500: 56% ± 4%; PEG 4000: 80% ± 4%; PEG 8000: 78% ± 2%; PEG 10,000: 85% ± 3%; PEG 12,000: 75% ± 7%; PEG 20,000: 77% ± 2%; Sorbitol 7 *w*/*v*%: 100% ± 2%; Sorbitol 10 *w*/*v*%: 100% ± 3%; Sorbitol 12.5 *w*/*v*%: 100% ± 3%; and Sorbitol 35 *w*/*v*%: 92% ± 3.6%; Levels of significance after statistical analysis treated cells compared against their respective untreated control group: PEG 200: ****; PEG 300: ****; PEG 400: ****; PEG 600: ****; PEG 1000: ****; PEG 1500: ****; PEG 4000: *; PEG 8000: ****; PEG 10,000: **; PEG 12,000: *; PEG 20,000: ****; Sorbitol 7 *w*/*v*%: ns; Sorbitol 10 *w*/*v*%: ns; Sorbitol 12.5 *w*/*v*%: ns; and Sorbitol 35 *w*/*v*%: ns.

Figure 2 shows that sorbitol solutions did not decrease the cell viability of treated Caco-2 cells according to the MTT assay. However, low molecular weight PEGs such as PEG 200, 300, and 400 severely reduced surviving cells. The other derivatives had less impact on cell viability, yet none of them had higher values than 85% and the results varied greatly regardless of molecular weight.

**Figure 3:**
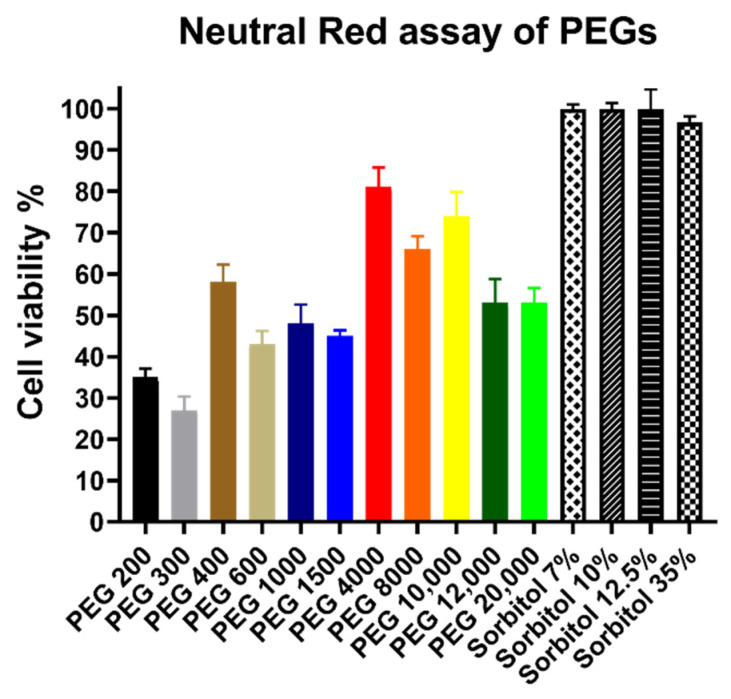
Cytotoxicity of PEG and sorbitol solutions measured by NR assay on Caco-2 cells. Concentrations of all PEG solutions were 30 *w*/*v*% and volumes were 100 µL. Cell viability expressed as the percentage of the absorbance of the untreated control cells. Columns represent the mean, ± SEM, n = 10. Cell viability of the samples (mean ± SEM): PEG 200: 35% ± 2%; PEG 300: 27% ± 3%; PEG 400: 58% ± 4%; PEG 600: 43% ± 3%; PEG 1000: 48% ± 5%; PEG 1500: 45% ± 1%; PEG 4000: 81% ± 5%; PEG 8000: 66% ± 3%; PEG 10,000: 74% ± 6%; PEG 12,000: 53% ± 6%; PEG 20,000: 53% ± 4%; Sorbitol 7 *w*/*v*%: 100% ± 1%; Sorbitol 10 *w*/*v*%: 100% ± 1%; Sorbitol 12.5 *w*/*v*%: 100% ± 5%; and Sorbitol 35 *w*/*v*%: 97% ± 1%; Levels of significance after statistical analysis treated cells compared against their respective untreated control group: PEG 200: ****; PEG 300: ****; PEG 400: ****; PEG 600: ****; PEG 1000: ****; PEG 1500: ****; PEG 4000: **; PEG 8000: ns; PEG 10,000: ns; PEG 12,000: **; PEG 20,000: ****; Sorbitol 7 *w*/*v*%: ns; Sorbitol 10 *w*/*v*%: ns; Sorbitol 12.5 *w*/*v*%: ns; and Sorbitol 35 *w*/*v*%: ns.

Neutral Red assay, as seen in Figure 3, revealed nearly identical trends compared to the MTT assay. The sorbitol solutions had no cytotoxic effects. Low molecular weight PEGs had higher impact on cell viability, however the difference between high and low molecular weight derivatives is smaller. The results of high molecular weight derivatives also had severe deviation.

### 3.3. Autophagy Assay Results

**Figure 4:**
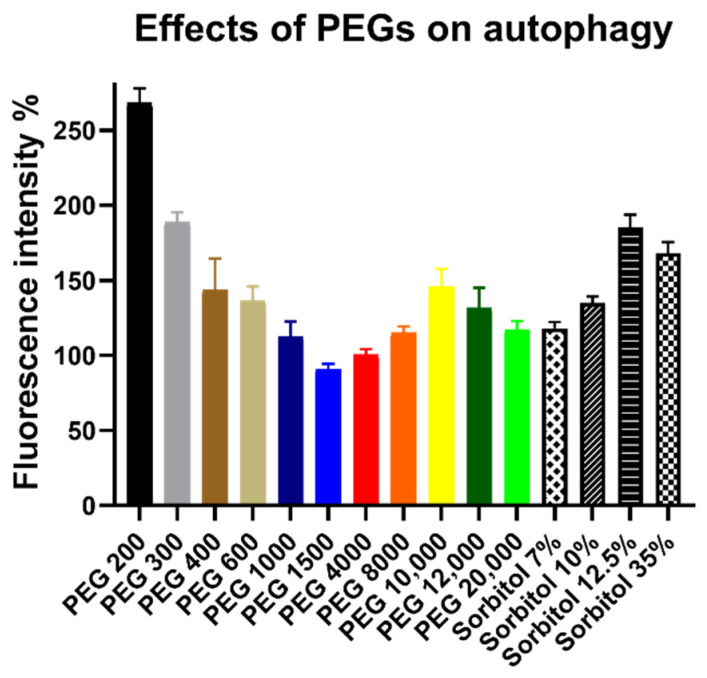
Effect of PEG and sorbitol solutions on the number of autophagosomes. Concentrations of all PEG solutions were 30 *w*/*v*% and volumes were 100 µL. Fluorescence intensity is expressed as the percentage of the intensity of the untreated control cells. Columns represent the mean, ± SEM, n = 4, after the highest and lowest values were excluded. Fluorescence intensity of the samples (mean ± SEM): PEG 200: 268% ± 10%; PEG 300: 189% ± 6%; PEG 400: 144% ± 21%; PEG 600: 136% ± 10%; PEG 1000: 113% ± 10%; PEG 1500: 91% ± 4%; PEG 4000: 101% ± 4%; PEG 8000: 115% ± 4%; PEG 10,000: 146% ± 12%; PEG 12,000: 132% ± 13%; PEG 20,000: 117% ± 6%; Sorbitol 7 *w*/*v*%: 118% ± 4%; Sorbitol 10 *w*/*v*%: 135% ± 5%; Sorbitol 12.5 *w*/*v*%: 185% ± 8%; and Sorbitol 35 *w*/*v*%: 168% ± 7%.

Nearly all treatments increased the number of autophagosomes, as can be seen on Figure 4. From PEG 200 to PEG 1500, there was a decreasing trend of fluorescence intensity which changed after PEG 1500 and started increasing again. Sorbitol solutions had nearly similar results than their PEG counterparts with the exception of 12.5 *w*/*v*%, which had higher results than PEG 1000. PEG 200 had a drastically higher intensity than any other treatment as the rest of the chemicals had their results in the range of ~180% to 100%.

### 3.4. Flow Cytometry Results

**Figure 5:**
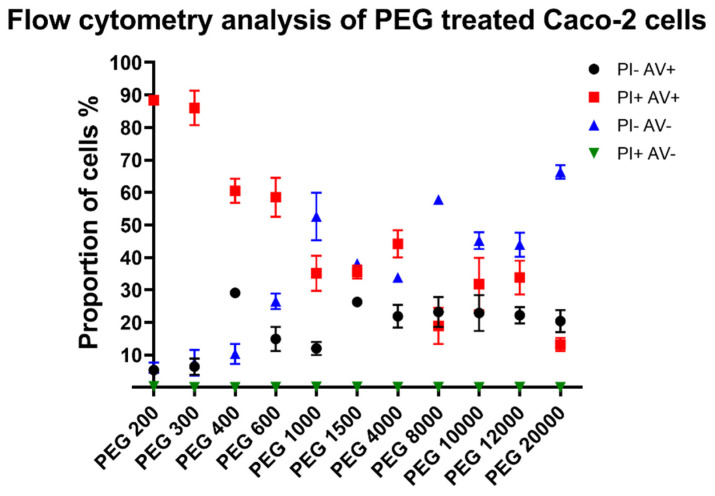
Proportion of living, dead, and apoptotic Caco-2 cells, measured by flow cytometry, treated with 1 mL of 30 *w*/*v*% solutions of PEGs, stained with propidium iodide (PI) and annexin V (AV). Each point represents the mean of triplicates. Data represented as percentage distribution of cells between necrotic (PI+ AV+), early apoptotic (PI− AV+), living (PI− AV−), and incorrectly stained (PI+ AV−) populations of three independent experiments. Distribution of the samples as mean ±SEM (PI− AV+; PI+ AV+; PI− AV−; and PI+ AV−): PEG 200: 5.3% ± 1.3%; 88.4% ± 0.6%; 6% ± 1.6%; and 0.3% ± 0.3%; PEG 300: 6.4% ± 2.5%; 86% ± 5.3%; 7.6% ± 4%; and ±0% ±0%; PEG 400: 29.1% ± 0.6%; 60.5% ± 3.7%; 10.3% ± 3.1%; and ±0% ±0%; PEG 600: 14.9% ± 3.7%; 58.5% ± 6%; 26.5% ± 2.4%; and 0.1% ± 0.1%; PEG 1000: 12% ± 2%; 35.1% ± 5.4%; 52.6% ± 7.3%; and 0.2% ± 0.1%; PEG 1500: 26.3% ± 0.7%; 35.5% ± 2%; 38.1% ± 1.2%; and 0.2% ± 0.1%; PEG 4000: 21.9% ± 3.5%; 44.2% ± 4.2%; 33.8% ± 0.8%; and 0.1% ± 0%; PEG 8000: 23.2% ± 4.6%; 18.9% ± 5.6%; 57.8% ± 1.1%; and 0.1% ± 0.1%; PEG 10,000: 22.9% ± 5.5%; 31.8% ± 8.1%; 45.2% ± 2.6%; 0% ± 0%; and PEG 12,000: 22.2% ± 2.5%; 33.8% ± 5.2%; 43.9% ± 3.7%; and 0.1% ± 0.1%.

As sorbitol solutions had only limited impact on cells, we excluded them from further experiments. For flow cytometry, Caco-2 cells were stained with propidium iodide and labelled with annexin V to distinguish between necrotic and early apoptotic cells. Figure 5 shows the distribution of gated cells. The proportion of unstained, living cells (PI− AV −) increases with molecular weight. For low molecular weight PEGs, necrotic cells (PI+ AV+) are the dominant for dead cells and but with the increase in molecular weight the ratio of early apoptotic cells (PI− AV+) increases.

### 3.5. In Vivo Toxicity Test

**Figure 6:**
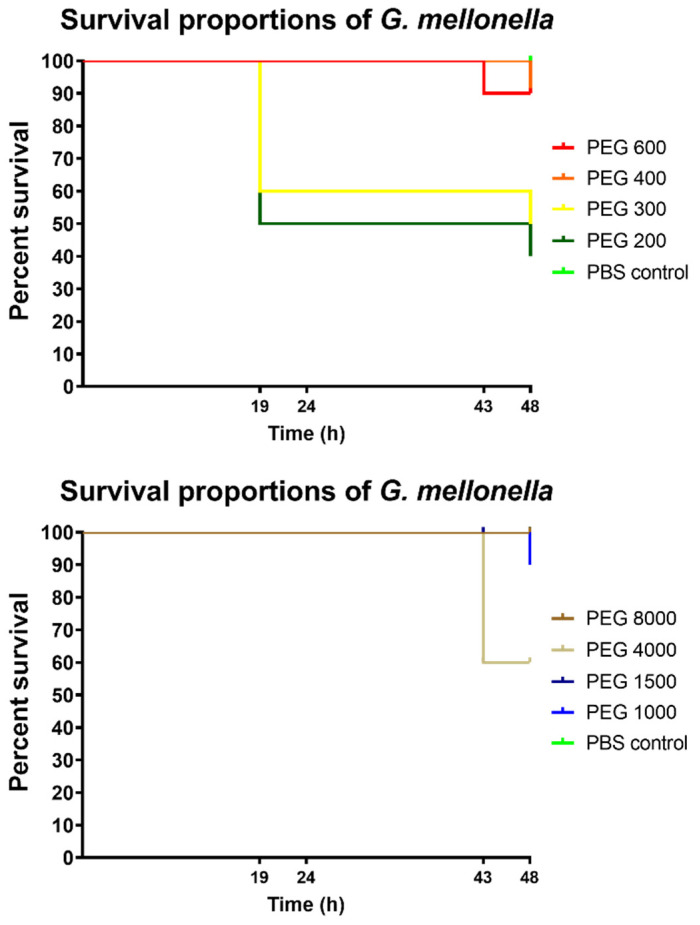

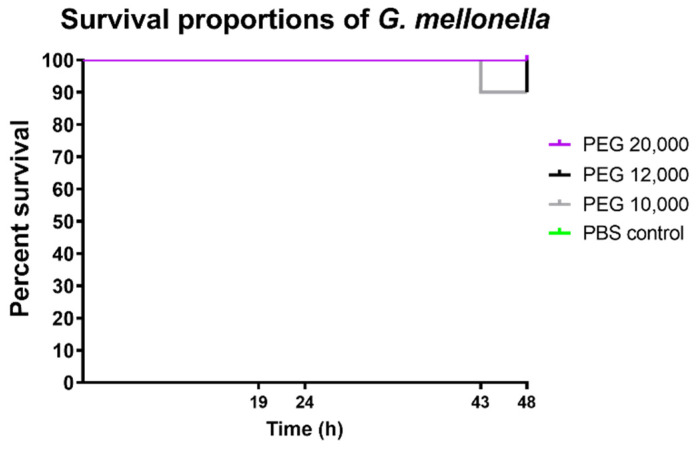
Kaplan–Meier survival curves of *G. mellonella* larvae. Each group consisted of 10 specimens, who were injected with 20 µL of test samples. The following death events occurred during the experiment: 19 h: PEG 200: 5 (top), PEG 300: 4 (top); 43 h: PEG 600: 1 (top), PEG 4000: 4 (middle), PEG 10,000: 1 (bottom); 48 h: PEG 200: 1 (top), PEG 300: 1 (top), PEG 400: 1 (top), PEG 1000: 1 (middle) PEG 12,000: 1 (bottom). Significance of treated groups compared to PBS control according to Mantel–Cox log-rank test, and Gehan–Breslow–Wilcoxon test: PEG 200: **/** (top); PEG 300: */* (top); and PEG 4000: */* (middle); all others were insignificant.

*G. mellonella* larvae were injected with 20 µL of the PEG solutions, their viability was checked four times during the two-day long experiment. The treated groups consisted of 10 healthy specimens. Figure 6 shows that mortality was significant for low molecular weight PEGs 200 and 300 (top) and medium molecular weight PEG 4000 (middle). No other PEG decreased survival of larvae significantly.

### 3.6. Correlation of Results with Molecular Weight

As it can be seen in Table 1, we used Spearman correlation to find statistical relationship between the different measured data and the average molecular weight of PEGs. Osmolality, the proportion of necrotic cells (PI+ AV+ cells according to the flow cytometry) and total larvae mortality significantly decreases as molecular weight increases. The ratio of living cells according to both cytotoxicity assays and flow cytometry escalates as the PEGs average molecular weight increases. However, autophagy and the proportion of apoptotic cells had no statistical relationship with the chemical structure of PEGs.

Additionally, the correlation matrix was calculated to compare the different data with each other, which is shown in Table 2. Autophagy had no statistical relationship overall. The other molecular weight independent cellular effect, the proportion of early apoptotic cells, had negative correlation with larvae mortality and a positive correlation with the NR assay. The different cell viability measurements (MTT, NR assays, and proportion of necrotic and living cells according to flow cytometry) had significant correlation with each other and with osmolality. The last data set, total larvae mortality, was only connected with flow cytometry results. Naturally, the most significant correlation could be found between the distribution of necrotic and living cells at flow cytometry.

## 4. Discussion

Polyethylene glycols referred to as Macrogols in the European Pharmacopoeia [3], are one of the important excipients used in oral or parenteral dosage forms. Primarily, PEGs are clinically used as primary constipation treatment or to prepare for colonoscopy [16,17]. Due to their outstanding characteristics, such as chemical neutrality, hydrophilicity, high and moderate solubility in aqueous and organic solvents, respectively, and flexibility of backbone chain, these polymers are widely applied to improve pharmacological and biological properties of different pharmaceutical formulations [18]. PEGs are well-known for use in the development of stealth coating of nanoparticle surfaces which is based on the interaction between opsonins and the PEGylated surface [19,20]. As such, both physical properties and cellular effects of PEGs are important, yet most publications focus only on one given PEG and studies involving multiple derivatives are limited in number. We aimed to investigate their cellular effects on a wide scale of molecular weight and calculate correlation between the different measured properties. Previously, it was stated that simple biological effects such as cytotoxicity of PEGs are based on osmolality and can be predicted from molecular weight [21].

By first measuring osmolality and later investigating cytotoxicity, autophagy, necrotic/apoptotic/living cell distribution, and in vivo toxicity, we tried to study multiple aspects of PEGs’ biological effects. We used an OSMOMAT 070 osmometer for the measurement of osmolality of our solutions as vapor pressure method is appropriate for determination of osmolality or molecular weight [22,23,24,25]. In vitro cytotoxicity experiments were carried out on Caco-2 human colorectal adenocarcinoma cells as a general model cell line, but also as indicators of the susceptibility of the gastrointestinal tract as they morphologically represent the intestinal epithelium [26,27]. The cytotoxicity was assessed by MTT and Neutral Red assays which are widely used in vitro techniques. These methods complement each other as MTT detects cell viability based on an enzymatic conversion, while on the other hand, cellular uptake and incorporation of neutral red is measured. Due to the different mechanism of action, several publications applied both methods to investigate cytotoxicity of different chemicals [28,29,30]. In order to further study cytotoxic properties of PEGs, we used labelled annexin V and propidium iodide to discriminate between necrotic and early apoptotic cells with flow cytometry [29,31,32]. Autophagosome formation was measured by cellular organelle staining as a marker of autophagy, an inducible mechanism of cytotoxicity [33,34,35]. The different cytotoxic experiments were supplemented with the use of *Galleria mellonella* injection method. It is an emerging in vivo model organism for the determination of toxicity of various xenobiotics [36,37,38]. The method shows good correlation with cellular and other in vivo techniques in case of acute toxicity studies [39].

Regarding cytotoxicity, previously, few articles investigated the cytotoxic properties of PEGs on a wide scale of molecular weight. Liu et al. studied a series of PEG derivatives on the HeLa (human cervical cancer cells) and the L929 cell line (fibroblasts derived from mice) with MTT assay [40]. The involved PEG derivatives were TEG (triethylene glycol), PEG oligomers (PEG 400, PEG 1000, PEG 2000, and PEG 4000), and PEG-based monomers PEG methyl ether acrylate (mPEGA) and PEG methyl ether methacrylate (mPEGMA-500 and mPEGMA-950)). Their research showed that the PEG 400 and PEG 2000 were almost non-cytotoxic at a 5 mg/mL concentration. However, PEG 1000 and PEG 4000 were more toxic to cells, especially to the L929 cell line. This indicates that molecular weight and thus osmolality are not connected to cytotoxicity. Contrary to their report, we found that the cytotoxicity levels of PEG 400, PEG 1000, and PEG 4000 are ascending according to the MTT assay. The protocol of MTT assay is different between the two experiments, as we treated the cells with a much higher concentration of PEGs for a shorter period while Lie et al. incubated the cells for 24 h. The latter highlights the influence of PEGs on the proliferation of the cells while our protocol was more focused on the acute effects. Additionally, Hodaei et al. reported that Caco-2 cells after 24 h of incubation of 4 *w*/*v*% PEG 4000, PEG 6000, PEG 10,000, and PEG 35,000 no cytotoxic effect could be observed (cell viability: 100%, 96%, 92%, and 88%, respectively) while PEG 400 and PEG 15,000 at 4 *w*/*v*% exhibited significant toxicity on cells (cell viability 45% and 48%, respectively) as measured by MTT assay [12]. These results confirm that the acute, high concentration treatment has a significantly different pattern, than a long-term incubation as cell proliferation and cytotoxicity are not influenced in the same way by the presence of PEGs. Postic et al. investigated the effect of PEG 200, 2000, and 20,000, and poly(vinyl pyrrolidone) 8000 on membrane transport apoptotic markers, cell viability by PrestoBlue assay, cell morphology, and caspase 3/7 activity on metastatic melanoma A375, mouse fibroblast 3T3, and human corneal epithelial cells [41]. After 72 h of incubation, higher concentrations of low molecular weight PEGs significantly changed the number and morphology of A375 cells compared to PVP 8000. Resazurin assay indicated that PEG 200 was more cytotoxic to A375 and 3T3 cells than the other PEGs, and PVP 8000 had a unique dose-dependent killing action against the cells. However, in the case of human corneal epithelial cells, all the three PEGs were significantly different from each other. Caspase 3/7 activation showed time dependency and no correlation with molecular weight. Overall, it must be noted that the different cellular effects of PEGs were highly influenced by the incubation time and the type of cells as well.

Taken together, Spearman correlation showed (Table 1), that the average molecular weight of studied PEGs had significant positive correlation with cell viability according to MTT (*p* = 0.0055) and NR assays (*p* = 0.0444) and the proportion of living cells according to flow cytometry (*p* = 0.0018). Additionally, a negative correlation was found between molecular weight and osmolality (*p* = 0.0039) and proportion of necrotic cells (*p* = 0.0001). This clearly means that low molecular weight PEGs have the highest cytotoxic activity, and this effect decreases with the increase in average PEG chain length. According to the correlation matrix, osmolality can be correlated with MTT (*p* < 0.0001), NR (*p* = 0.0018), proportion of living (*p* = 0.0182), and necrotic cells (*p* = 0.0098). These results further confirm that cytotoxicity of PEGs could be explained by the strong osmotic shock (30 *w*/*v*% solutions of PEGs, incubation time: 30 min) they inflict upon the cells. However, sorbitol solutions showed no cytotoxicity in the case of the MTT and NR assays, despite the fact that their osmolality was comparable to PEGs. We assume that osmolality alone cannot be responsible for the observed cell viability decrease and a not investigated factor or cellular effect, which is linked with molecular weight, must be responsible for the cytotoxicity of PEGs. Wang et al. reported that the mechanism of cellular uptake of PEGs was dependent on the molecular weight, as low molecular weight PEGs (750–2000) were taken up only by passive diffusion while, for longer derivatives, endocytosis was also an important mechanism [26]. Cellular uptake was also influenced by the time of incubation and temperature. Mesenchymal stem cells tolerated sodium chloride, sorbitol, and PEG 3000 in significantly different ways, although they were applied at the same osmolality [42]. The type of osmolyte influenced numerous investigated factors of chondrogenesis regardless of osmolality. As such, a possible explanation for our results is that only low molecular weight PEGs could enter the cells during the short incubation time and the specific sensitivity of Caco-2 cells towards PEGs and their tolerance towards sorbitol.Unfortunately, there are no publications regarding the effects of non-modified PEGs in *G. mellonella* larvae and on autophagosome formation, thus we cannot compare our results with previous publications. However, the relationship of osmotic stress and induction of autophagy is well studied and shows a strong influence [43,44]. Autophagosome formation had no correlation with molecular weight, which can be explained by recent findings, which indicate that generally, autophagy is a late phase response to osmotic stress [44]. The high autophagosome formation can be explained by the easy passage of low molecular weight PEGs into cells as it was reported, that even 30 min of hyperosmotic incubation can lead to formation of proteosomes [45]. Additionally, further investigation is required to determine which characteristic of PEGs is the most influential in the process. Surprising cellular effects of PEGs are not unheard of, as it was reported that PEG 35 can influence uptake of exosomes and reduce the levels of IL-1β [46]. Unmodified PEGs were previously not injected into *G. mellonella* for evaluation of toxicity, and we have found that only molecular weight (*p* = 0.0403) had significant correlation with total larvae mortality. Macromolecules are rarely tested in this new model organism, yet in high concentration they show concentration dependent toxicity [47]. Labelled annexin V was used as an indicator of early stage apoptosis, and we have found that the proportion of stained cells had no correlation with osmolality. The literature reports that linking PEG 12,000 to interferon-α2b did not significantly increase apoptotic activity, as well as PEG 5000 coated silver nanoparticles compared to citrated coated ones [48,49]. Further flow cytometry experiments are needed to describe how cells are divided between necrotic and early apoptotic population after PEG treatment, as we suspect that it is heavily influenced by PEG concentration and incubation time.

It can be concluded that osmolality and cell viability (measured by different methods) had significant correlation with molecular weight of PEGs, while the more complicated effects, such as early stage apoptosis, autophagosome formation, and larvae survival (in vivo toxicity) are not directly linked with molecular weight. When it is needed to predict biological effect of a previously untested PEG, only simple cellular interactions, such as (cyto)toxicity (through necrosis), can be estimated based on previous experiments with different molecular weight derivatives. Further studies are needed to reveal what properties of PEGs influence more complex mechanisms such as autophagosome formation or apoptosis. Through detailed testing, the responsible factors can be identified, and statistical correlations can be found to prove relations between biological effects and chemical or physical attributes. Until then, we suggest caution with estimation of cellular action of PEGs purely based upon molecular weight when a new derivative is compared to known ones.

## 5. Conclusions

In summary, various cellular effects, in vivo toxicity, and osmolality of eleven different polyethylene glycols were studied on Caco-2 cells. Statistical analysis of the various data sets found a significant correlation of cytotoxicity, osmolality, and molecular weight. Overall, low molecular weight PEGs exhibited significant decrease in cell viability and high osmolality and larvae mortality, while high molecular weight usually had limited effect on cells and decreased osmolality. Further scientific investigations should be undertaken to discover more about the effects of PEGs on autophagosome formation and early apoptosis, however it can be stated that certain biological action of PEGs cannot be estimated based on molecular weight.

## Figures and Tables

**Table 1:** Correlation of measured data (only PEGs) with average molecular weight calculated by Spearman method. Significance levels are shown as: ns = *p* ≥ 0.05; * = *p* < 0.05; ** = *p* < 0.01 and *** = *p* < 0.001.

	Spearman Correlation Coefficient	Level of Significance
**Osmolality**	−0.8091	**
**Cell viability—MTT**	0.7909	**
**Cell viability—NR**	0.6241	*
**Autophagy**	ns	
**Proportion of PI** **−** **AV+ cells**	ns	
**Proportion of PI** **−** **AV+ cells**	−0.9273	***
**Proportion of PI** **−** **AV− cells**	0.8455	**
**Total larvae mortality**	−0.6357	*

**Table 2:** Correlation of matrix of all measured data sets (only PEGs). Spearman correlation coefficient and significance levels are shown as: ns = *p* ≥ 0.05; * = *p* < 0.05; ** = *p* < 0.01; and **** = *p* < 0.0001.

	Osmolality	Cell Viability—MTT	Cell Viability—NR	Autophagy	Proportion of PI− AV+ Cells	Proportion of PI+ AV+ Cells	Proportion of PI− AV− Cells	Total Larvae Mortality
**Osmolality**	-	0.909/****	−0.843/**	ns	ns	0.755/*	−0.709/*	ns
**Cell viability—MTT**	−0.909/****	-	0.770/**	ns	ns	−0.736/*	0.664/*	ns
**Cell viability—NR**	−0.843/**	0.770/**	-	ns	0.620/*	ns	ns	ns
**Autophagy**	ns	ns	ns	-	ns	ns	ns	ns
**Proportion of PI** **−** **AV+ cells**	ns	ns	0.620/*	ns	-	ns	ns	−0.626/*
**Proportion of PI+ AV+ cells**	0.755/*	−0.736/*	ns	ns	ns	-	−0.973/****	0.771/**
**Proportion of PI** **−** **AV** **−** **cells**	−0.709/*	0.664/*	ns	ns	ns	−0.973/****	-	−0.771/**
**Larvae mortality**	ns	ns	ns	ns	−0.626/*	0.771/**	−0.771/**	-

## Data Availability

All data available upon request from the corresponding author.
